# Supplementary material for: Variable phylosymbiosis and cophylogeny patterns in wild fish gut microbiota of a large subtropical river
Source: mSphere. 2025 Mar 28;10(4):e00982-24. doi: 10.1128/msphere.00982-24 (PMC12039269; doi:10.1128/msphere.00982-24)
Supplement: Supplemental material — Table S1 caption; Table S2; Figures S1-S8. [file msphere.00982-24-s0001.pdf]

Supplementary materials

Supplementary Table 1 Metadata of the ecological and sampling information of the fish species for statistical analysis.
(Supplementary files. Table S1.xlsx)

Supplementary Table 2 The variance of fish gut microbiota composition explained by environmental factors in the lower research of the Pearl River

Environment factor	R^2	P-value
TEM	0.0864	0.001
pH	0.0188	0.172
DO	0.1629	0.001
TN	0.1822	0.001
TP	0.1337	0.001
Conductivity	0.0022	0.795
TDS	0.1357	0.001
SD	0.1088	0.001
Chla	0.166	0.001

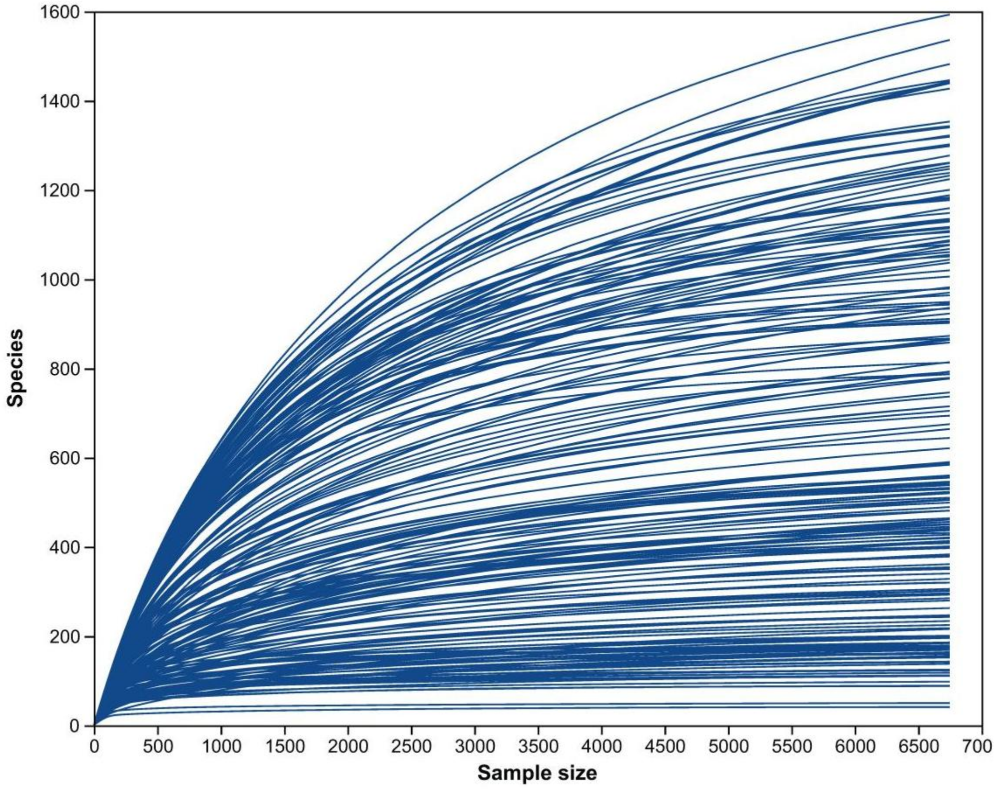

Supplementary Figure 1: rarefaction curves showing the correlation between sequencing depth and bacteria taxa recovered for all gut samples sequenced.

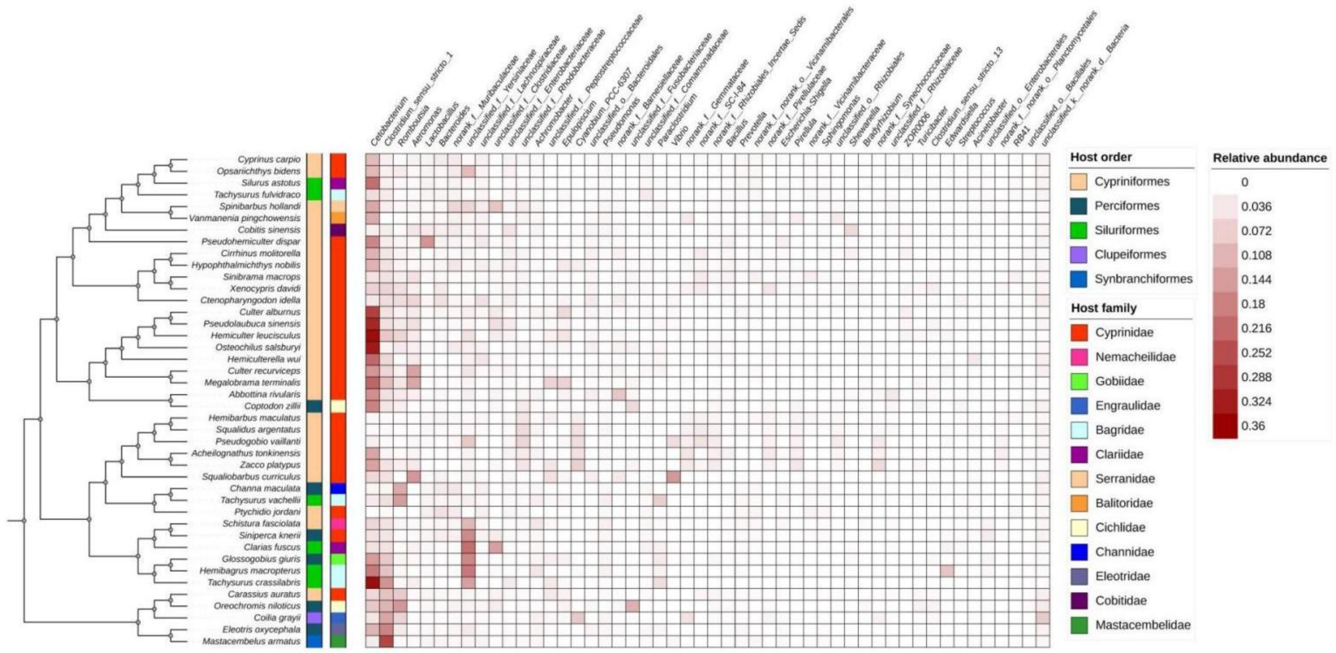

Supplementary Figure 2: Heatmap indicated relative abundance of the top 50 bacterial genus in each of the fish species. Microbiota dendrogram according to mean of bray-curtis distances among different species

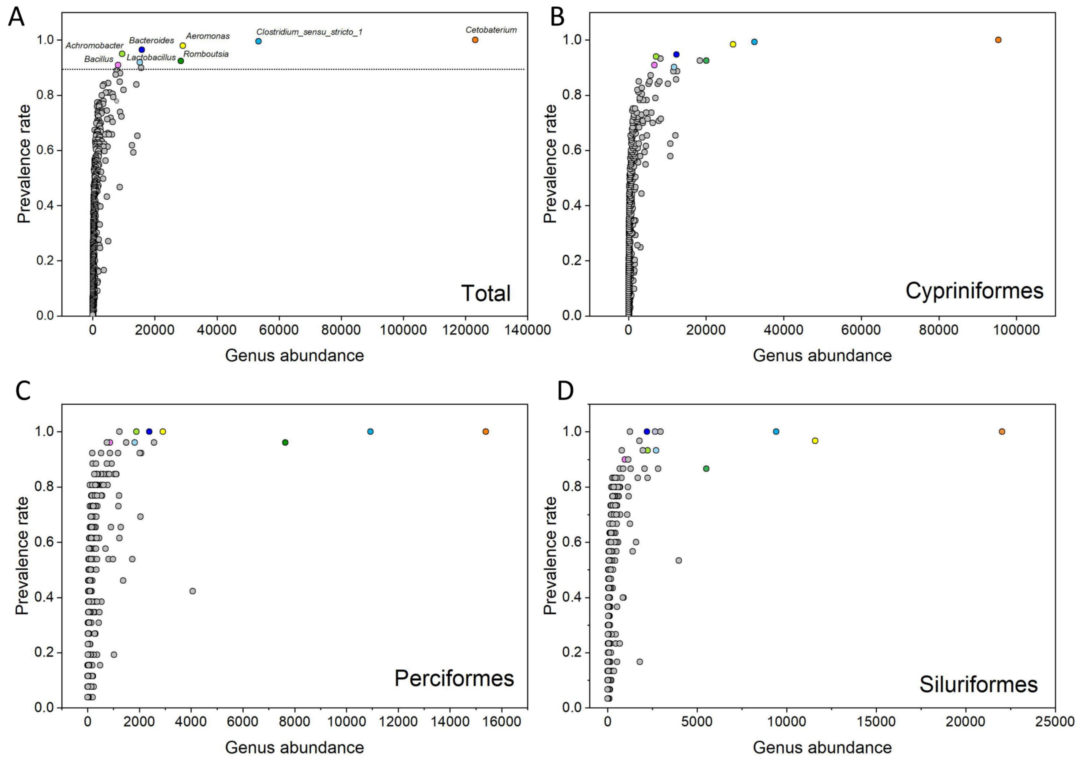

Supplementary Figure 3: The eight mostly prevalent genera (*Cetobaterium*, *Clostridium_sensu_stricto_1*, *Aeromonas*, *Romboutsia*, *Bacteroides*, *Lactobacillus*, *Achromobacter*, *and Bacillus*) are shown in samples from three different orders (All, Cypriniformes, Perciformes, Siluriformes)

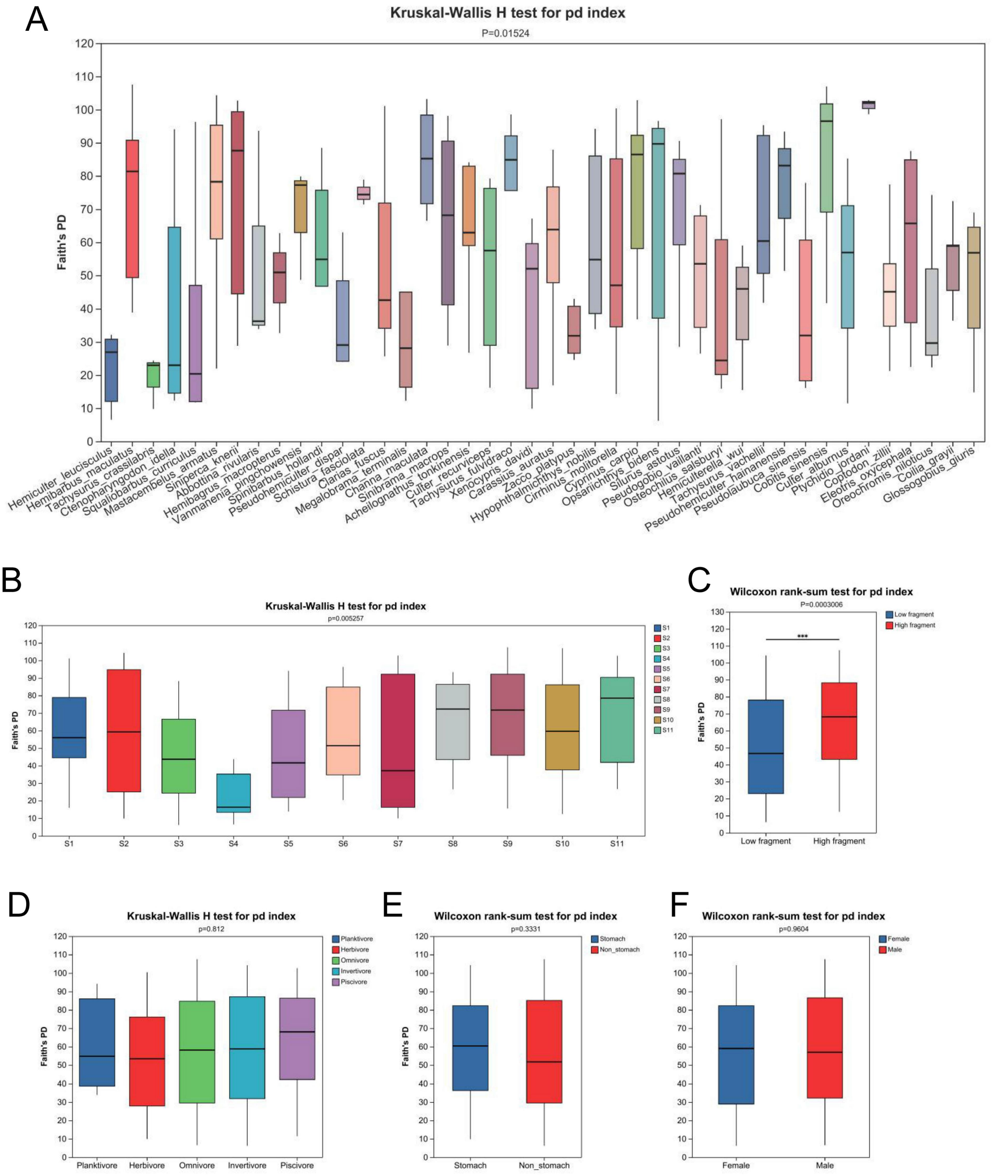

Supplementary Figure 4: α-diversity of fish gut microbiota in hosts of different species,habitat type, geographical location, dietary group, and stomach type

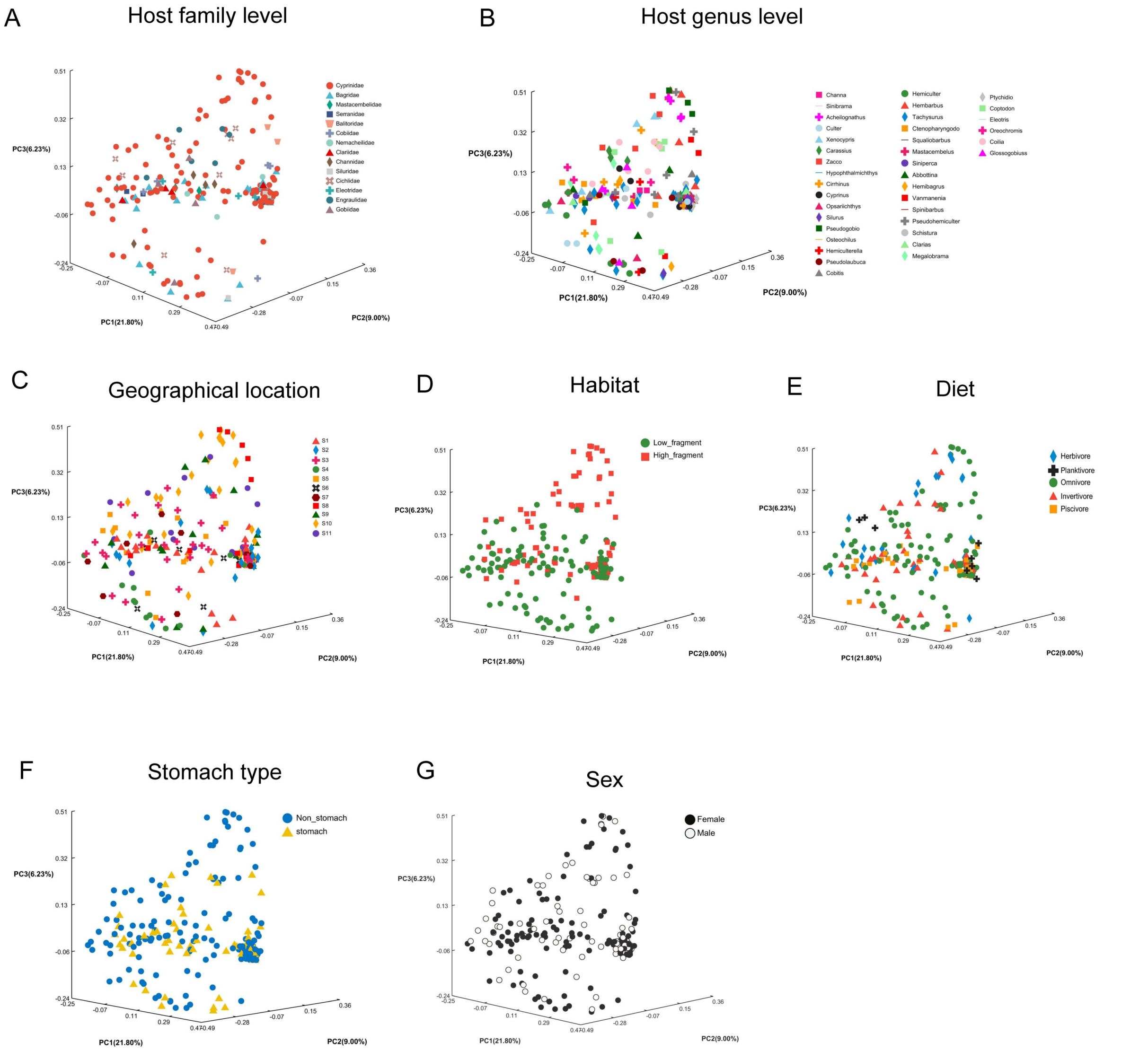

Supplementary Figure 5: PCoA plots based on Bray-Curtis dissimilarity showing the variability in microbial composition between fish gut microbiome samples. Colour and shape of different data points corresponds to the (A) host family; (B) host genus; (C) geographical locations; (D) habitat; (E) dietary preference of host; (F) stomach type; (G) sex

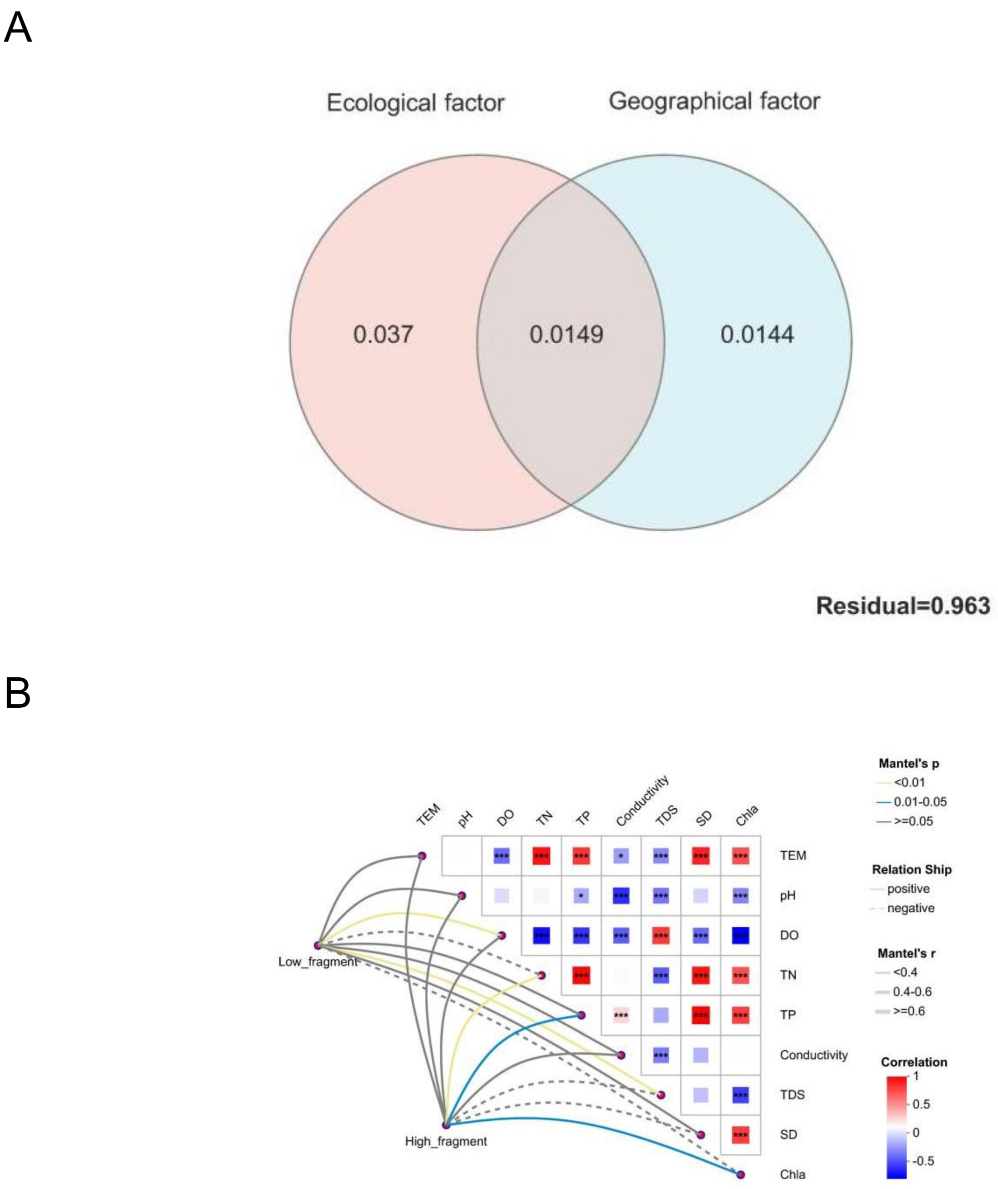

Supplementary Figure 6: (A) Variation partitioning of environmental factors and geographical distance; (B) Relationship between environmental factors and fish gut bacterial community. Asterisks represent P-values: ***$P < 0.001$; **$P < 0.01$; *$P < 0.05$.

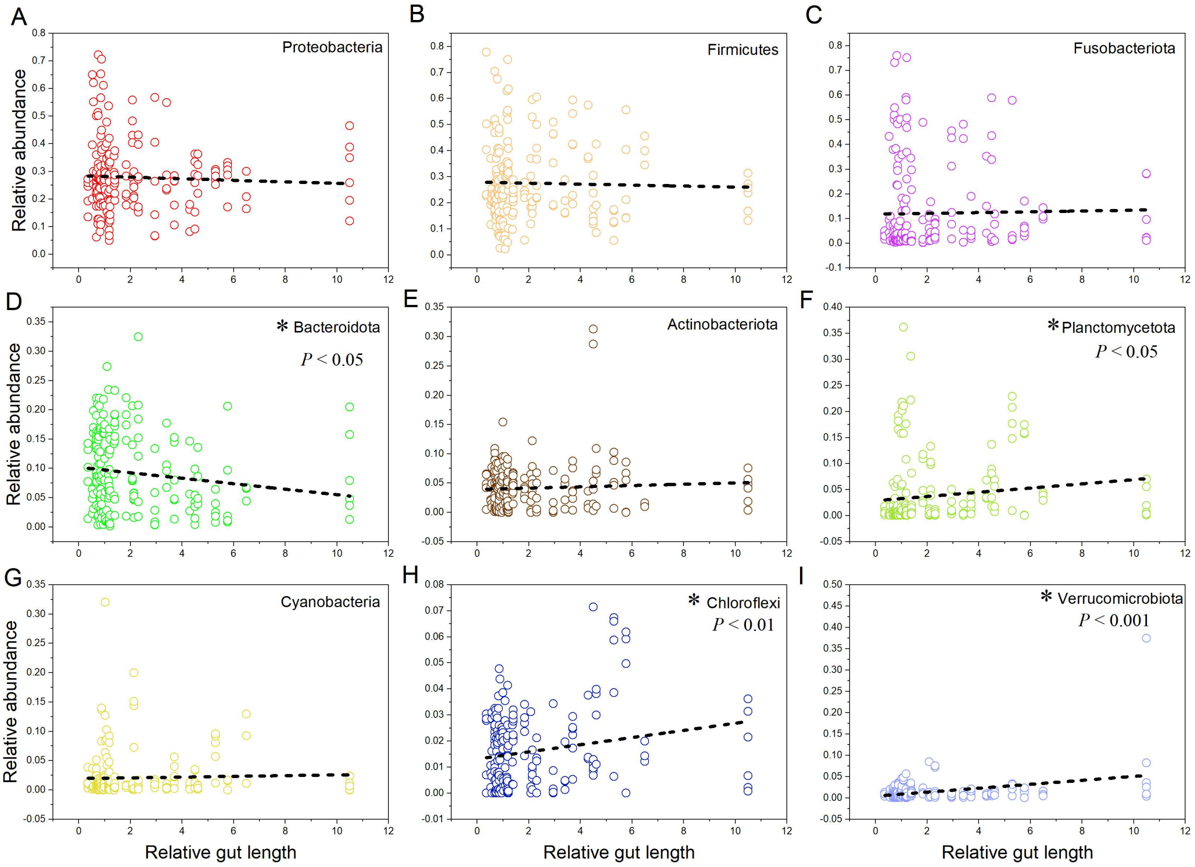

Supplementary Figure 7: Linear regression of relative abundance of gut core microbiome (the phylum level) among all fish species vs. their relative gut length, (A) Proteobacteria; (B) Firmicutes; (C) Fusobacteriota; (D) Bacteroidota; (E) Actinobacteriota; (F) Planctomycetota; (G) Cyanobacteria; (H) Chloroflexi; (I) Verrucomicrobiota.

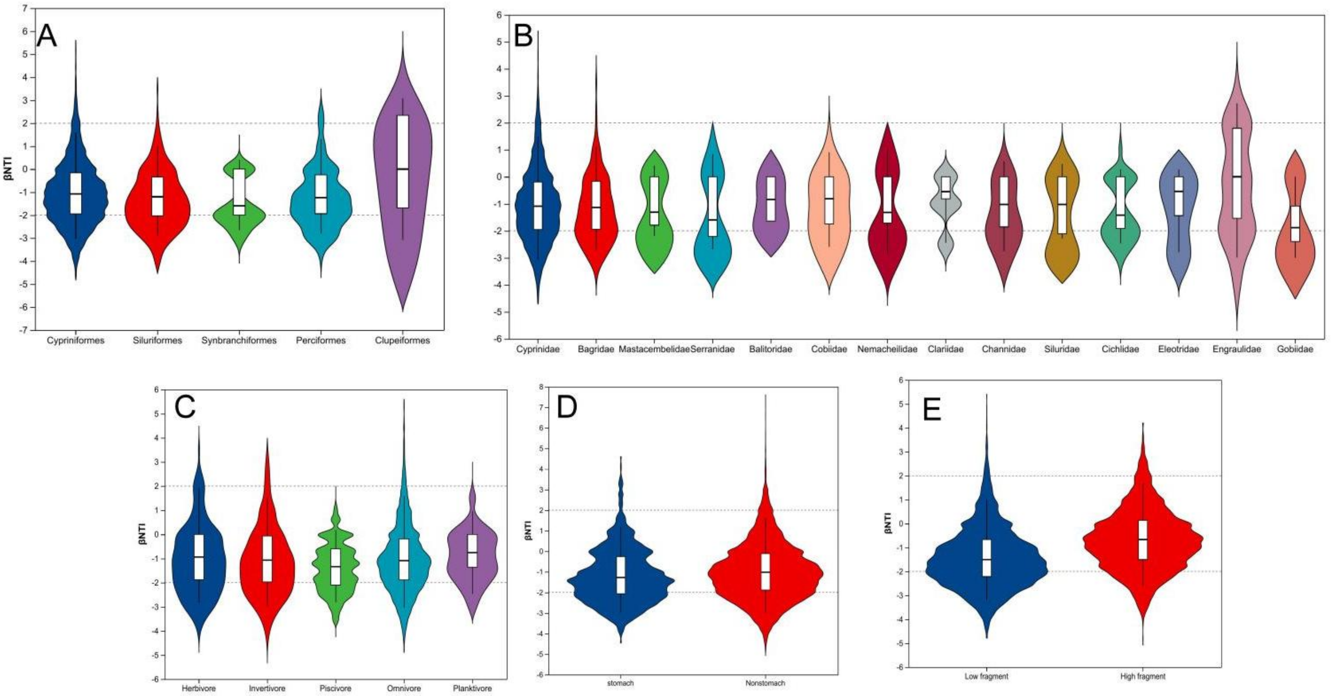

Supplementary Figure 8: Null model was used to analyze the assembly process of gut microbial community in hosts of different species (A,B), dietary group (C), stomach type (D) and habitat type (E).
